# The Rise and Fall of Physiological Theories of the Control of Human Eating Behavior

**DOI:** 10.3389/fnut.2022.826334

**Published:** 2022-05-17

**Authors:** David A. Levitsky, Laura Barre, John Jeshurun Michael, Yingyi Zhong, Yitong He, Alyse Mizia, Sahib Kaila

**Affiliations:** ^1^Division of Nutritional Sciences, Cornell University, Ithaca, NY, United States; ^2^Department of Psychology, Cornell University, Ithaca, NY, United States

**Keywords:** physiological signals, eating behavior, meal initiation, meal termination, theories of eating

## Abstract

Kuhns was the first to suggest that theories in science do not develop in small increments but rather in major leaps to paradigms that examine the same question through very different perspectives. Theories on the mechanism responsible for control of human food intake fall into Kuhn’s description. This article describes how the two major theories of the control of food intake in humans, the Glucostatic Theory, and the Lipostatic Theory, showed initial promise as explanations, but later deteriorated with the slow accumulation experimental data. The locus of theories considered eating behavior as a part of physiological system that regulates the storage of energy on the body. We challenge this fundamental belief with data which suggests that we must be ready to accept a major change in the way we think about eating behavior if we are ever to decrease the prevalence of obesity.

## Introduction

**FIGURE 1:**
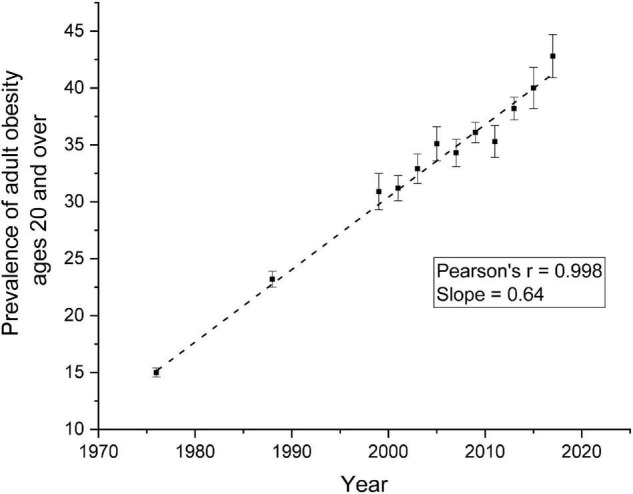
Prevalence of Overweight, Obesity, and Severe Obesity Among Adults Aged 20 and Over: United States, 1960–1962 Through 2017–2018 (74).

Thomas Kuhns proposed in his famous book, *The Structure of Scientific Revolution* (1), that science does not advance in small incremental steps. Rather, science leaps from well-accepted paradigms to very different ones that demand new methodologies, new analytic techniques, and new ways of the thinking. Research on the control of human food intake may be at that cusp where new perspectives are needed to help us answer one of our most serious public health issues, obesity. Figure 1 shows the prevalence of obesity over the past 40 years among adults United States. It is clear that obesity is linearly related to years, indicating our dismal failure to develop successful techniques to prevent and/or treat obesity.

This increase in body weight is a severe threat to public health because of the close association increased body weight and multiple chronic diseases including diabetes, cardiovascular disease, hypertension, and certain cancers that account for significant population morbidity, mortality, and expense (2). It is generally accepted that most of the increased weight is due to an increased caloric intake rather than a decrease in energy expenditure (3, 4). The daily increment in energy intake that can account for the increase in body weight of the population is very small. It is estimated to be between consuming only 25 and 150 kcals per day per year, an amount so small that we cannot accurately measure it (5).

The scientific responsibility for finding solutions to this rise in obesity has traditionally fallen on the fields of psychology and physiology. Both fields have enjoyed a long, albeit lopsided, contest to provide the best explanation for eating behavior and overeating. Such an explanation could potentially lead to the development of effective long-term therapies that would decelerate the insidious rise in body weight that accrues with age.

From the very beginning of the search for the determinants of eating behavior, physiological explanations have dominated the research. The discovery of the Horsley-Clarke stereotaxic in 1907, an instrument that allowed the fairly precise localization of brain structures, caused the flooding of the psychological literature with physiological studies searching for the “hunger” or “satiety” centers in the brain. By the late 1960s, the fruit of this marriage between psychology and physiology appeared to peak with the localization of the lateral area of the hypothalamus (LH) as the feeding center and the ventromedial nuclei of the hypothalamus (VMH) as the satiety center of the brain (6). From this apex of physiological research into the cause of eating behavior, two physiological theories emerged, the Glucostatic and the Set-Point theory of the control of food intake. Although each theory emphasizes different physiological signals that activate and deactivate eating behavior, both share the common believe that physiology drives the eating behavior. Both theories have similar histories based on “hard” physiological evidence. Both theories have since fallen. As Kuhns pointed out, the accumulated evidence could not explain the accumulating evidence. It is hoped that the illustration of the rise and fall of each of these theories will challenge us to reassess the role that physiology plays in determining human eating behavior.

### The Rise and Fall of the Glucostatic Theory of Eating Behavior

One of first physiological, and most tested, theory of the physiological control of food intake concerns the simple carbohydrate, glucose. It was based on the well-established fact that of the three storage depots for energy in the body (protein, fat, and carbohydrate), carbohydrates are the most plastic. More importantly, by the 1950’s the physiological mechanisms response for the regulation of blood were well established. It is a small step to suggest that eating behavior may be an extension of this regulatory system that maintains blood glucose constant.

For most humans, eating occurs a regular basis. When eating doesn’t occur, the concentration of blood glucose decreases. After eating a meal, glucose levels rise at a rate proportional to the carbohydrate content of the food consumed. Bulatao and Carlson (7) were one of the first to link the level of circulating glucose to eating behavior, albeit using dogs and measures of gastric motility as an indicator of hunger. The pioneering work and the originator of the Glucostatic Theory of Eating^1^ was Jean Mayer (9). Mayer was heavily influenced by his father, Andre Mayer, and his work on the control of body weight through variations in energy intake and expenditure in the rabbit (10, 11). One important concept he inherited from his father is the differentiation between short-term from long-term feeding mechanisms. Although Mayer focused on the role that glucose played in determining short-term food intake, he did attempt to bridge the gap between short term glucostatic mechanisms with the longer term “lipostatic” mechanisms being proposed by Kennedy and others (12).

Mayer initially reasoned that concentration blood glucose was a good condidate to be the stimulus that initates and terminates eating behavior. However, he was quite aware of the problem of explaining the eating behavior of the untreated diabetic. If eating behavior was inhibited by high levels of blood glucose, wouldn’t the untreated diabetic always feel satiated? Mayer’s answer was ingenious. The brain was not sensing the level of circulating blood glucose, but rather the utilization of glucose^2^. The untreated diabetic has high circulating glucose levels, but most of that glucose is not taken into cells and metabolized because of a simple lack of insulin (Type I diabetes) or disfunction of insulin receptors (Type II diabetes).

Mayer postulated that the brain sensed the lack of the utilization of glucose as the signal to eat and the utilization of glucose to cause satiety. As such, when one hasn’t eaten for several hours, blood glucose is low, but because there is no insulin, so the glucose does not enter cells to be metabolyzed. This causes eating to occur. On the other hand, after eating a meal, there is a rapid influx of glucose from the food into the blood, causing an increase in the release of insulin. This allows the glucose from the blood into cells, where utilization of that glucose would cause satiety. The untreated diabetic overeats because the lack of insulin limits the entry of glucose into cells and it’s utilization for energy.

Since the glucostatic theory so well-defined the conditions that initiate and terminate eating behavior, the theory provoked an enormous amount of research to test its validity. One of first tests of the theory in humans was performed by one of Mayer’s postdoctoral researchers, Ted van Itallie (14). By measuring the difference in arterial and venal blood to and from the arm of participants before and after eating, van Itallie et. demonstrated that eating occurred when the difference was small, and ceased when the differences were greater, i.e., after eating a meal. Even more impressive was a demonstration by Smith and Epstein (15) that an injection of 2-deoxyglucose, a substance that blocks intracellular glucose oxidation, will initiate eating in satiated rats.

Further evidence in support of the Glucostatic Theory to explain eating behavior emanated from studies of a substance call goldthioglucose. Marshall and Mayer (16) demonstrated than injections of goldthioglucose causes in neural damage in ventromedial area of the hypothalamus and produced hyperphagia and obesity in rats. What is important is that the toxic compound (gold) did not cause obesity if it was incorporated into either goldthiomalate or sodium thioglucose, as these compounds do not interfere with carbohydrate metabolism. Its relationship to the Glucostatic Theory became more clear when it was found not to produce obesity in diabetic mice (17) and was restored with insulin (18). These findings not only supported the idea that glucose utilization was important for the control of food intake and the regulation of body weight, but also helped establish the idea that the hypothalamus, unlike the rest of the brain, required insulin for glucose to enter the brain.

The analysis of the effects of hormones known to affect glucose metabolism seemingly added further support for the Glucostatic Theory. Glucagon, a hormone whose major function is to raise blood glucose levels and prevent hypoglycemia, when injected into humans, initially was found to significantly reduce food intake (19). This is exactly as expected, since the high glucose level would increase glucose utilization, causing satiety. Unfortunately, when the time course of the anorectic effect of glucagon was scrutinized more closely, the time course did not synchronize with the change in glucose utilization. The increase in glucose utilization begins approximately 30 min after injection, but the inhibition of food intake is delayed, peaking about 2 to 3 h later, and continues for about 5 h (20).

A similar uncoupling between the action of a hormone on glucose metabolism and its effect on food intake occurred with the investigation of insulin. The primary function of insulin is to reduce blood glucose by increasing the uptake of glucose into peripheral cells and increasing its utilization to produce ATP. According to the Glucostatic Theory, these events should be the ideal conditions to cause satiation. Early studies of the effects of insulin injections failed to find any effect on food intake. However, a study by MacKay and Callaway (21) demonstrated that the use of “standard” insulin had little effect on eating behavior, but large increases in food intake, eventually leading to obesity. In experimental animals, obesity could be obtained using protamine zinc injections, a form of insulin that produced sustained decreases in blood glucose. This finding, of course, was opposite of what would be expected – insulin increases glucose utilization and should cause a decrease in food intake.

In humans, the evidence linking eating behavior to glucose utilization is equally as contradictory. Infusing glucose into humans causes an increase in glucose utilization, but does not cause a decrease in food consumption (22, 23). An even clearer demonstration that there is a lack of effect from circulating glucose on food intake was provided by studies utilizing the euglycemic clamp. This procedure allows the assessment of blood glucose independent of blood insulin. Here again, food intake was not related to blood glucose or to glucose utilization (24, 25).

One of the most contradictory effects of the Glucostatic Theory of eating behavior occurs just prior to initiating feeding. First discovered by Louis-Sylvestre and Le Magnen (26) in free-feeding animals, then confirmed in free-feeding humans (27), glucose utilization increases just prior to eating a meal. According to the Glucostatic Theory, one should be satiated just before eating. Obviously, such a theory could not stand the test of time.

Thus, although the Glucostatic Theory of Eating control of food intake was a creative and integrative attempt to understand eating behavior within the realm of the regulation of blood glucose, in the end it failed as it could not explain accumulating evidence obtained by more precise measurements.

### The Rise of the Set-Point Theory and the Triumph of Leptin

Gordon Kennedy, a British physiologist, revolutionized the way we think about brain feeding centers and body weight. In a few simple yet elegant studies, Kennedy demonstrated that those hypothalamic “centers” do not directly control eating behavior. Instead, they are part of a larger regulatory system involved in the regulation of body fat (weight). Kennedy proposed that the hypothalamus acted as a “lipostat” that monitors the amount of peripheral body fat, compared that value to a predetermined (genetic) value, then translated the difference into feeding behavior (28, 29). If the amount of body fat was less than the programmed value, the hypothalamus would elicit eating behavior. If the signal from the periphery indicated more than the expected value, then eating behavior would be inhibited. Although Kennedy never used the term, the conceptual framework that Kennedy described has been referred to as the “Set-Point” theory of regulation of body weight.

It took about 10 years for the Set-Point theory of feeding behavior to gain wide acceptance in both psychology and physiology. The idea of a single set-point expanded into multiple set points allowing it could “explain” a wide range of behaviors such as seasonal fluctuations in eating behavior and body weight (30) and estrus cycling of eating and body composition in female mammals (31). The Set-Point theory could also justify the dismal failure to produce an effective therapeutic method to help people produce a sustained reduction in body weight. No matter how hard one worked at losing weight, it would return to the pre-dieting level (32) apparently driven by the forces operating through the control of energy intake and expenditure to maintain the “set-point” for body weight (33). An avalanche of research followed, with major discoveries of brain circuits and neurochemicals linking the various brain loci to the behavior of eating (34).

One critical aspect of the Set-Point theory that remained unresolved for approximately 40 years was the identification of the signal that transmitted information about peripheral fat stores to the brain. Such a signal had to (a) originate in adipose tissue, (b) be released into the blood in an amount proportional to the size of the fat depot, and (c) activate the brain mechanisms involved in eating behavior^3^. Many putative substances were proposed to play this role including circulating progesterone concentration (35) and blood glycerol (36), but none seemed to hold up to the scrutiny of scientific investigation.

In 1994, Jeffrey Friedman’s group reported that they had identified the messenger and named it leptin (37). They created a mouse model with a genetic mutation that prevented the mouse from producing leptin: a small protein synthesized in adipose cells. The result was that mice were hyperphagic, hypometabolic, and obese compared to their lean counterparts without the mutation. Impressively, leptin fit beautifully into Kennedy’s Set-Point theory of the regulation of body weight. The absence of this signal would be read by the hypothalamus as an indication that the body did not have sufficient fat stores. In response to the low levels of leptin, the hypothalamus of these mutant mice would initiate eating and decrease metabolic rate to restore the amount of body fat to its normal level. These mice were obese because their adipose cells were incapable of producing leptin, resulting in an increase in eating behavior and a decrease in metabolic rate.

**FIGURE 2:**
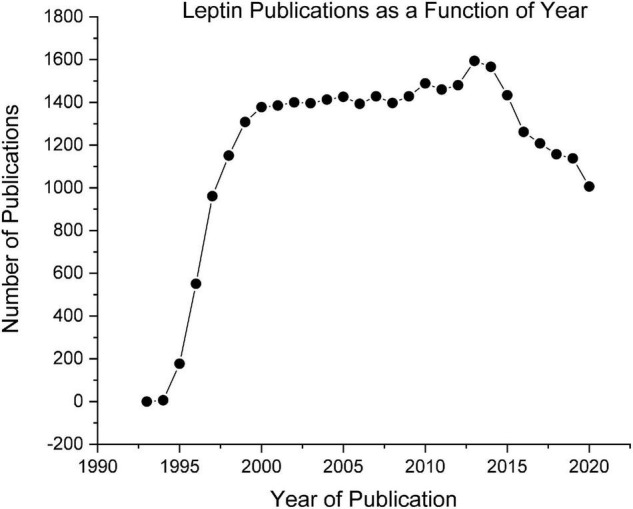
Leptin publications as a function of year obtained from PubMed.

The conceptualization of leptin as a regulator of body weight fits perfectly into the Set-Point theory and was overwhelmingly accepted by the vast majority of researchers including those working in the field of obesity. As seen in Figure 2, soon after the initial publications of leptin an explosion of research followed, attempting to establish connections between the leptin receptors and the brain neurochemicals that were directly activating and inhibiting eating behavior such as neuropeptide Y, agouti-related peptide, and proopiomelanocortin. The pinnacle of success of the leptin and the Set-Point theory of body weight regulation was reached in 1995, when three articles appeared simultaneously in the journal *Science*, demonstrating that the daily injection of leptin into these genetically leptin-deficient obese mice caused (a) a reduction in food intake, (b) an increase in metabolic rate, and (c) a normalization of body weight (38–40).

The acceptance of leptin as the signal from adipose tissue to the brain facilitated the acceptance of the Set-Point theory of the control of body weight throughout the fields of physiology and psychology. This discovery provided hope that regulating leptin synthesis might be possible to stem the tide of the increasing prevalence of overweight individuals and obesity. The pharmaceutical industry would merely have to produce a leptin analog and find a way to deliver it. The brain would read this message as an indication that the body contained more fat than the hypothalamic mechanisms indicated was necessary, which would suppress eating behaviors and increase metabolic rate, allowing the person to lose weight without feeling hungry. Even better for the pharmaceutical industry, people would have to take the leptin analog their whole life; once an individual stopped, they would return to their previous weight. Pharmaceutical companies would financially benefit from this opportunity as well as clinicians and individuals with obesity looking for solutions that did not require voluntary behavioral change.

Psychologists succumbed to using physiological techniques and the language of physiology to investigate and explain the behavior of eating and the phenomenon of obesity. Using animal models exclusively, colorful maps of various areas in the brain were published illustrating the many neurochemicals that caused either an increase or a decrease in the behavior of eating. Psychology conferences were filled with slides of brain anatomy and terminology of neuroanatomical structures. The confrontation of an increasingly recognized problem of the growing “obesity epidemic” was being led by biologists.

### The Decline of the Set-Point Theory

Despite the almost universal acceptance of the Set-Point theory of weight control, primarily due to the discovery of leptin as the possible messenger between peripheral adipose tissue and the brain, small cracks in the theory began to emerge in the literature. The first was the discovery that, unlike the mice, humans that were obese did not produce less leptin than those that were non-obese, but rather produced more leptin (41). This seemingly contradictory finding to the leptin theory of body weight regulation was quickly overcome by (a) the demonstration that leptin concentration was proportional to the degree of adiposity, a necessary component of the Set-Point theory (42) and (b) a slight modification of the Set-Point explanation of obesity, which could be made with the assertion that human obesity was not caused by a lack of the signal (leptin) but rather a lack of sensitivity of the brain receptors that received the signal (43).

Other weaknesses of the Set-Point theory, unfortunately, soon followed. Close scrutiny of the dynamics of leptin secretion in response to fasting and refeeding provided another problem for the Set-Point theory. During weight loss due to fasting, blood leptin decreased at a much faster rate than the reduction in fat mass (41) and returned to normal levels almost immediately upon re-feeding, considerably faster than the repletion of fat stores in adipose tissue (44). This dissociation between leptin secretion and amount of adiposity was not viewed as a major problem with the Leptin-Set-Point theory, but it did muddle the original straightforward interpretation of leptin as the messenger of peripheral adiposity to the brain.

Even more discouraging for those believing leptin was going to be the answer to the “obesity epidemic” was the limited number of people identified with a deficiency in leptin production. In 2008, a worldwide search identified only 12 patients diagnosed with leptin deficiency (45), hardly enough to explain the rising prevalence of obesity. Considering there are approximately 650 million adults with obesity in the world, leptin deficiency would account for an infinitesimal fraction of obesity. Still, for those individuals with leptin deficiency, leptin supplementation produced a miraculous reduction in their hunger and obesity (46, 47).

Another disappointment for the leptin theory of the regulation of body weight was the lack of response to supplemental leptin in overweight humans. In a study conducted by Lejeune et al. (48), a group of overweight men was given repeated daily leptin injections for 46 days. Despite raising leptin levels to more than 400 times the normal level, the men failed to show any significant change in the rate of weight loss, fat composition, lean body mass, or energy expenditure.

Leptin failed to fulfill the role as the unique messenger from peripheral adipose tissue to the brain. Without the identification of an accurate feedback signal from peripheral fat to the brain, the physiological Set-Point theory was critically threatened as the explanation of the control of food intake and regulation of body weight.

### The Emergence of Non-homeostatic Feeding Systems

Besides the dissolution of the Glucostatic Theory and the Lipostatic (Set-Point) Theory as viable explanations that control human food intake, other more recent challenges to the idea that eating behavior are driven by physiological mechanisms. At the heart of both the Glucostatic and the Lipostatic was the concept of homeostasis, a historic idea in physiology dating back to the work of Walter Cannon (49) at the beginning of the 20th century. Homeostasis, applied to eating behavior and the Set-Point theory of body weight, holds that some constituent of the body such as adiposity, lean body mass, and/or glucose is maintained constant through the control of eating behavior. The activation of eating behavior occurs because of a depletion of one or more of these critical constituents (fat, glucose, glycogen, etc.) and the cessation of feeding behavior results from the repletion of those critical physiological constituents.

Research began to reveal that besides homeostatic eating, the behavior of eating could be driven by non-homeostatic mechanisms such as taste or pleasure or past environmental associations (50, 51). This challenge was not initially a threat to the physiological determinant of feeding because most of the research that emerged highlighted the neurological substrates of non-homeostatic eating (52, 53). Moreover, the idea of non-homeostatic eating was embraced by the biologists because it seemed to expand the physiological control of eating to the more psychological areas of reward and motivation, needing and wanting, and overlapped with the research area of drug addiction (54).

A merger between the classic physiological neural substrates related to eating and the neural circuits involved in more psychological causes of eating behavior such as wanting and needing, seem to offered a more comprehensive understanding of human eating behavior (55). Nevertheless, this merger distracted most researchers in the field from the simple fact that if non-physiological stimuli elicited by learning or the environment could drive eating behavior, then the predictability of physiological mechanisms as the cause of eating would be limited.

### Is Body Weight Regulated?

People who strongly believe that human eating behavior is driven by physiological homeostatic mechanisms readily argue that the fact that long-term stability of weight persists even with large variations in daily energy intake and expenditure proves physiological regulation exists. The argument is as follows: the average American consumes about 880,000 kilocalories per year but gains only about one pound per year, which is equivalent to an excess of 3,500 kilocalories. If we consider the weight gain to be an energetic error, then this error is less than 1% (3,500 kcal/880,000 kcal). Does this not provide irrefutable evidence that humans possess a fairly precise physiological control of food intake?

This is not necessarily so. An alternative model can be constructed without the necessity of a physiological control over food intake or expenditure, as a similar model was suggested by Westerterp et al. (56). The model can be built on three simple but plausible assumptions: (1) daily consumption of energy intake is a random process having a normal distribution, (2) daily energy expenditure is also a random process having a normal distribution, and (3) energy expenditure increases or decreases proportionally to the changes in body weight. Such a system will maintain body weight relatively constant without the necessity of feedback to food intake or energy expenditure. Thus, the demonstration that the long-term stabilization of body weight can be observed despite daily fluctuations of energy intake and expenditure is not necessarily proof of the existence of physiological regulatory mechanisms.

**FIGURE 3:**
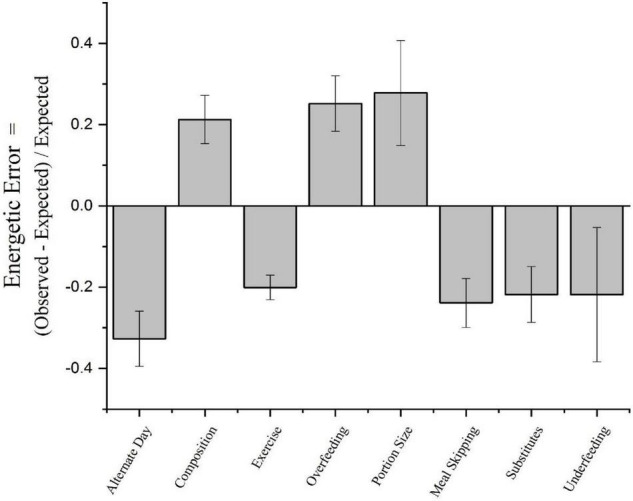
Energetic errors imposed by various methods. From Levitsky et al. (57).

Moreover, a close examination of the precision to which energy intake precisely compensates for changes in energy balances indicate that humans do a fairly poor job. To estimate the degree to which humans precisely compensate for imposed energetic challenges, we reviewed studies where humans were subjected to either an energy surplus or energy deficit and energy intake was measured (57). These challenges included: alternate-day fasting, changes in diet composition, exercise, overfeeding, changes in portion size, meal skipping, dietary manipulation with fat and sugar substitutes, and underfeeding. These data were extracted from 592 groups drawn from 200 studies consisting of a total of 13,203 participants. The energetic error was defined as: (observed energy intake – expected energy intake)/expected intake. As can be seen in Figure 3, in no case is the energetic error zero. Rather the energetic error is positive when a reduction in energy intake is required and negative when an increase in energy intake is needed to compensate for the energy imbalance. The mean absolute average energetic error is 24 percent. Given the average intake of Americans is about 2,900 kcal (57) per day, an energetic error of 24 percent represents approximately 700 kcal a day, which is more than sufficient to account for the secular increase in body weight observed in adults in the United States of about 2.2 pounds per year^4^. Thus, without compensating for caloric surfeits from being given larger portions of food with a higher caloric content than what we would eat at home, we will gain weight.

### Control Body Weight Independent of the Control of Food Intake

As the theoretical model described above suggests, it is possible to control body weight without the need to control food intake. There is little dispute that associated with weight loss due to a reduction in energy intake is a decrease in total energy expenditure. Moreover, an increase in weight gain as a result of overfeeding is associated with an increase in energy expenditure (58). Such changes in energy expenditure with changes in body weight are due to at least two factors. First, accompanying any change in body weight is a proportional change in lean body mass (59). Lean body mass constitutes about 33% of weight loss (59) and about 38% weight gain (60). Because lean body mass has a considerably higher metabolic rate than fat mass (61), the total metabolic rate will increase with weight gain and decrease with weight loss. The second factor is due to the physics of carrying weight. The larger the body weight, the great the energy expended to carry that weight. Both factors would allow a return of body weight to pre-existing levels following weight loss due to undereating (62) or overeating (63) without a change in food intake.

### Energy Intake Is Related to Lean Body Mass and Not to Fat Mass

Finally, central to the Set-Point Theory of the regulation of body weight is the idea that the brain is operating to maintain total amount of adipose tissue constant. Therefore, one would expect a negative correlation to be evident between total fat mass and *ad libitum* energy intake. When fat content is reduced then food intake should be stimulated, supposedly through the inhibition of leptin. However, several investigators have found little to no relationship between adipose mass and food intake. This lack of relationship is not due to poor measuring instruments because in its place highly significant correlations were found between lean body mass and food intake (64–71). The strong implication of these studies is that it is not adipose tissue that is regulated through the control of food intake, but rather it may be lean body mass. Currently, we do not know the signal that lean body mass use to communicated with the brain, but it appears to act more like a slow modulator of intake, rather than a determinant.

### What Is the Future of Physiological Control of Body Weight?

We are not arguing that the behavior of eating is independent of physiological processes. All behavior emanates from the brain. We do wish that we rethink the role that physiological processes play determining when and/or how much we eat at the next meal. Perhaps, re-examining the possible connections between lean body mass and eating behavior may lead to possible pharmacological interventions that may be safer and more effective means to suppress body weight for long periods of time. Indeed, the time course over which physiological signals affect eating behavior should be re-examined. Perhaps it is time to expand upon the classical view that the meal is the intersection where the physiological signals interact with behavior (72) and in its place we should consider the possibility that regulation of body weight occurs over longer time intervals such as days or even weeks (73). To begin to think about such an idea, we must change our expectations of rapid weight loss to one that is considerably slower and more realistic. Obesity does not develop overnight. It results from a slow yet insidious positive energetic error that accumulates with time. Rethinking physiological regulation over longer spans of time requires creative and imaginative ideas both in terms of what to measure and how to measure it for long periods of time. If we are ever to reduce that age-related weight gain curve (Figure 1), we must release our tenacious grasp on physiology as the answer to our obesity problems and have the courage to try new ideas. Perhaps, we are at a cusp where we must consider Kuhns’ suggestion, that a new paradigm in our understanding of the control of food intake and the regulation of body weight in humans.

## Author Contributions

DL conceived and created first draft. LB contributed to ideas and edited. JJM, YZ, and YH checked references and edited. AM critiqued draft and edited. SK edited the draft. All authors contributed to the article and approved the submitted version.

## Conflict of Interest

The authors declare that the research was conducted in the absence of any commercial or financial relationships that could be construed as a potential conflict of interest.

## Publisher’s Note

All claims expressed in this article are solely those of the authors and do not necessarily represent those of their affiliated organizations, or those of the publisher, the editors and the reviewers. Any product that may be evaluated in this article, or claim that may be made by its manufacturer, is not guaranteed or endorsed by the publisher.

## Footnotes

1 For an excellent history of the Glucostatic Theory of Eating see van Itallie (8).

2 Interestingly, Mayer’s theory predicted that the hypothalamus, unlike other brain tissue, must have insulin receptor to be able to sense glucose utilization. The discovery of insulin receptors in the hypothalamus was discover almost 20 years after Mayer’s introduction of the Glucostatic Theory later (13).

3 While both mechanisms involved in eating behavior and energy expenditure are involved in the regulation of body weight, this discussion will focus only the control of energy intake.

4 Assuming the average intake 2,900 kcal/day, a 24% error would amount to close to 700 kcal/day while the increment in caloric intake necessary to account a one lb. gain in weight is about 10 kcal per day (5).
